# Targeting Forward and Reverse EphB4/EFNB2 Signaling by a Peptide with Dual Functions

**DOI:** 10.1038/s41598-020-57477-x

**Published:** 2020-01-16

**Authors:** Chiyi Xiong, Yunfei Wen, Jun Zhao, Dengke Yin, Lingyun Xu, Anca Chelariu-Raicu, Cody Yao, Xiaohong Leng, Jinsong Liu, Rajan R. Chaudhari, Shuxing Zhang, Anil K. Sood, Chun Li

**Affiliations:** 1 Departments of Cancer Systems Imaging, The University of Texas MD Anderson Cancer Center, Houston, Texas 77054 United States; 2 Departments of Gynecologic Oncology and Reproductive Medicine, The University of Texas MD Anderson Cancer Center, Houston, Texas 77054 United States; 3 School of Pharmacy, Anhui University of Chinese Medicine, Hefei, 230012 P.R. China; 4 School of Biology and Pharmaceutical Engineering, Wuhan Polytechnic University, Wuhan, 430023 China; 5 Departments of Pathology, The University of Texas MD Anderson Cancer Center, Houston, Texas 77054 United States; 6 Departments of Experimental Therapeutics, The University of Texas MD Anderson Cancer Center, Houston, Texas 77054 United States; 7 Center for RNAi and Non-Coding RNA, The University of Texas MD Anderson Cancer Center, Houston, Texas 77054 United States

**Keywords:** Cancer, Ovarian cancer

## Abstract

The tyrosine kinase receptor EphB4 is frequently overexpressed in ovarian and other solid tumors and is involved in interactions between tumor cells and the tumor microenvironment, contributing to metastasis. Trans-interaction between EphB4 and its membrane-bound ligand ephrin B2 (EFNB2) mediates bi-directional signaling: forward EFNB2-to-EphB4 signaling suppresses tumor cell proliferation, while reverse EphB4-to-EFNB2 signaling stimulates the invasive and angiogenic properties of endothelial cells. Currently, no small molecule–based, dual-function, EphB4-binding peptides are available. Here, we report our discovery of a bi-directional ephrin agonist peptide, BIDEN-AP which, when selectively internalized *via* receptor-mediated endocytosis, suppressed invasion and epithelial-mesenchymal transition of ovarian cancer cells. BIDEN-AP also inhibited endothelial migration and tube formation. *In vivo*, BIDEN-AP and its nanoconjugate CCPM-BIDEN-AP significantly reduced growth of orthotopic ovarian tumors, with CCPM-BIDEN-AP displaying greater antitumor potency than BIDEN-AP. Both BIDEN-AP and CCPM-BIDEN-AP compromised angiogenesis by downregulating epithelial-mesenchymal transition and angiogenic pathways. Thus, we report a novel EphB4-based therapeutic approach against ovarian cancer.

## Introduction

EphB4 belongs to the Eph family, the largest family of membrane-bound receptor tyrosine kinases. Both EphB4 and its sole physiologically relevant ligand, ephrin B2 (EFNB2), are membrane bound, and their interaction initiates forward signaling through EphB4 and reverse signaling through EFNB2^1^. EphB4 is overexpressed in many solid tumors, including those of the ovary^2,3^, breast^4,5^, colon^6^, prostate^7,8^, and bladder^9^. High EphB4 expression is associated with shorter disease-free survival in patients with epithelial ovarian cancer^3,10^. Independent of interaction with EFNB2, EphB4 can be activated by ligand-independent pathways to promote proliferation and metastasis, as EphB4 mutants with mutated phosphorylation sites can still promote tumor cell growth and migration^11^. Conversely, increasing evidence supports the idea that ligand-dependent EFNB2/EphB4 forward signaling is tumor suppressive. For example, EFNB2-Fc–mediated EphB4 phosphorylation activated Abl family tyrosine kinase and Crk adaptor protein in tumor cells, which led to significant tumor-suppressive activity^11–15^.

EFNB2 is expressed primarily on the surface of endothelial cells of tumor vasculature^16^. Several groups have shown that the reverse EphB4 to EFNB2 signaling in endothelial cells plays an essential role in initiating angiogenesis and lymphangiogenesis, important processes in supporting tumor growth and metastasis^17–19^. The soluble extracellular domain of EphB4, which disrupts the EphB4-EFNB2 interaction, attenuated angiogenesis and inhibited tumor growth^20^. EFNB2 functions as an important regulator of angiogenesis through activation of vascular endothelial growth factor receptor (VEGFR)^18^. Therefore, EphB4 agonists that activate the forward EphB4 signaling and interfere with the reverse EFNB2 signaling may offer a unique therapeutic opportunity.

While searching for stable EphB4-binding peptides with high binding affinity through structural modifications to the known EphB4 antagonist TNYLFSPNGPIARAW (TNYL-RAW)^21,22^, we unexpectedly discovered a high-affinity bi-directional ephrin agonist peptide (herein referred as BIDEN-AP) that selectively activated EphB4. Strikingly, BIDEN-AP differed from the EphB4 antagonist TNYL-RAW peptide by only a single amino acid, in that the L-Tyr in the P3-position of TNYL-RAW was replaced by a D-Tyr residue. Functional studies showed that BIDEN-AP could simultaneously activate EphB4-initiated Abl/Crk1 tumor-suppressive signaling in tumor cells and inhibit EFNB2-regulated angiogenic signaling in endothelial cells. Notably, systemic delivery of BIDEN-AP and its conjugate with core-crosslinked polymeric micelles (CCPM) displayed robust antitumor activity in orthotopic epithelial ovarian cancer models. Collectively, our studies support further development of this approach.

## Results

### Substitution of L-Tyr with D-Tyr in TNYL-RAW peptide led to conversion from EphB4 antagonist to EphB4 agonist

We used surface plasmon resonance (SPR) to measure the binding affinities of a small library of TNYL-RAW analogues to purified EphB4. Our data revealed that truncation of C-terminus Trp-P15 and Ala-Trp (P14-P15) motifs progressively reduced the binding affinity of peptides to EphB4 (Supplementary Data Fig. S1). This agrees with previous reports that the RAW motif is critical for fitting into the EFNB2 binding pocket of EphB4^23^. However, the N-terminus Thr-P1 and Thr-Asn (P1-P2) motif could be truncated without affecting the binding affinity of peptides, suggesting that the two N-terminal amino acids Thr-Asn are not directly involved in receptor binding and therefore could be used as a site for conjugation of imaging probes or pharmacokinetic modifiers in diagnostic and therapeutic applications.

Next, we performed a D-amino acid scan and discovered that substitution of Phe-P5 or Ser-P6 with their corresponding D-amino acids substantially decreased the binding affinity of the resulting peptides to EphB4. Substitution of Pro-P10 or Ile-P11 and double substitution of Pro-P7/Pro-P10 or Ala-P12/Ala-P14 with the corresponding D-amino acids completely abolished the binding affinity of the resulting peptides to EphB4 (Supplementary Data Fig. S2). These data indicate that these P5 to P12 amino acids contain important contact points with the EphB4 receptor and are needed for maintaining high receptor binding affinity.

**Figure 1:**
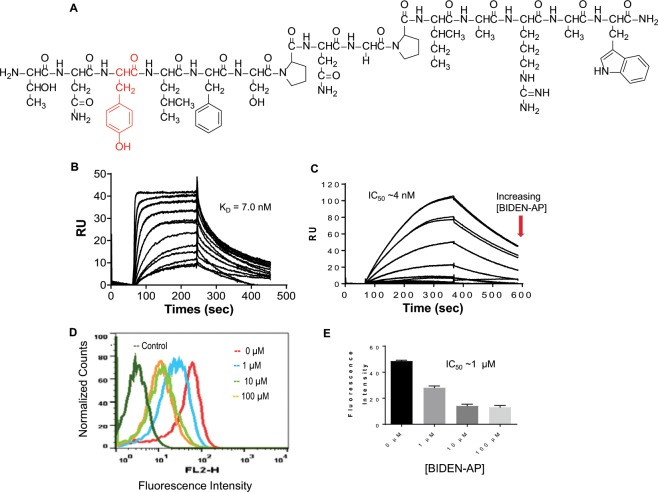
Novel EphB4 agonist BIDEN-AP has high receptor binding affinity. (**A**) Structure of BIDEN-AP. D-Tyr-P3 is highlighted in red. (**B**) Surface plasmon resonance (SPR) sensorgrams of BIDEN-AP. Vertical axes, in response units (RU), represent binding of the peptide to immobilized EphB4. (**C**) Competition of BIDEN-AP with EFNB2-Fc for EphB4 binding. BIDEN-AP at concentrations from 2 nM to 1000 nM was mixed with human EphB4 (30 nM) and injected onto sensor chips coated with EFNB2-Fc. (**D**) Competition of BIDEN-AP with EFNB2-Fc for EphB4 binding at the cellular level. A2780cp20 cells were co-incubated with EFNB2-Fc (20 nM) and BIDEN-AP (at concentrations from 0 to 100 µM) at 25 °C for 1 h. EFNB2-Fc bound to the cells was probed with phycoerythrin-labeled anti-Fc antibody and analyzed by flow cytometry. (**E**) Bound EFNB2-Fc as a function of BIDEN-AP concentration. Individual duplicates were performed in each condition. Data are presented as mean ± SD.

While studying the functional activity of TNYL-RAW analogues with high EphB4 binding affinity, we discovered that one such analogue, TNd(Y)LFSPNGPIARAW, in which the L-Tyr-P3 was substituted by a D-Tyr, induced EphB4 phosphorylation in human HeyA8 ovarian cancer cells (Fig. 1A, Supplementary Data Fig. S3, Table S1). Designated as BIDEN-AP, this peptide showed rapid association followed by slow dissociation from EphB4-coated sensor chips in the SPR assay^24^. The dissociation constant (K_D_) was estimated to be 7.0 nM after fitting of the datasets to a 1:1 mass transfer interaction model (Fig. 1B). The interaction relationship of BIDEN-AP and EFNB2 with EphB4 was studied by SPR, in which serial dilutions of BIDEN-AP were mixed with a fixed concentration of EphB4 and injected onto EFNB2-Fc–coated CM-5 chips. Sensorgrams show that the amount of EphB4 that bound to EFNB2-Fc decreased with increasing BIDEN-AP concentration. The median inhibitory concentration of BIDEN-AP (IC_50_) was estimated to be 4 nM (Fig. 1C). Thus, BIDEN-AP inhibited EphB4-EFNB2 interaction with high potency. At the cellular level, BIDEN-AP could effectively displace the binding between EFNB2-Fc and EphB4 on the surface of A2780cp20 cells, with an IC_50_ value of ~1 µM (Fig. 1D,E). The higher IC_50_ value in the cell-based assay than in the protein-based assay reflects the fact that EFNB2-Fc also binds to other Ephrin B class receptors^25,26^ and thus could not be completely displaced by BIDEN-AP. Collectively, these data indicate that BIDEN-AP and EFNB2 share the same EphB4 binding site.

**Figure 2:**
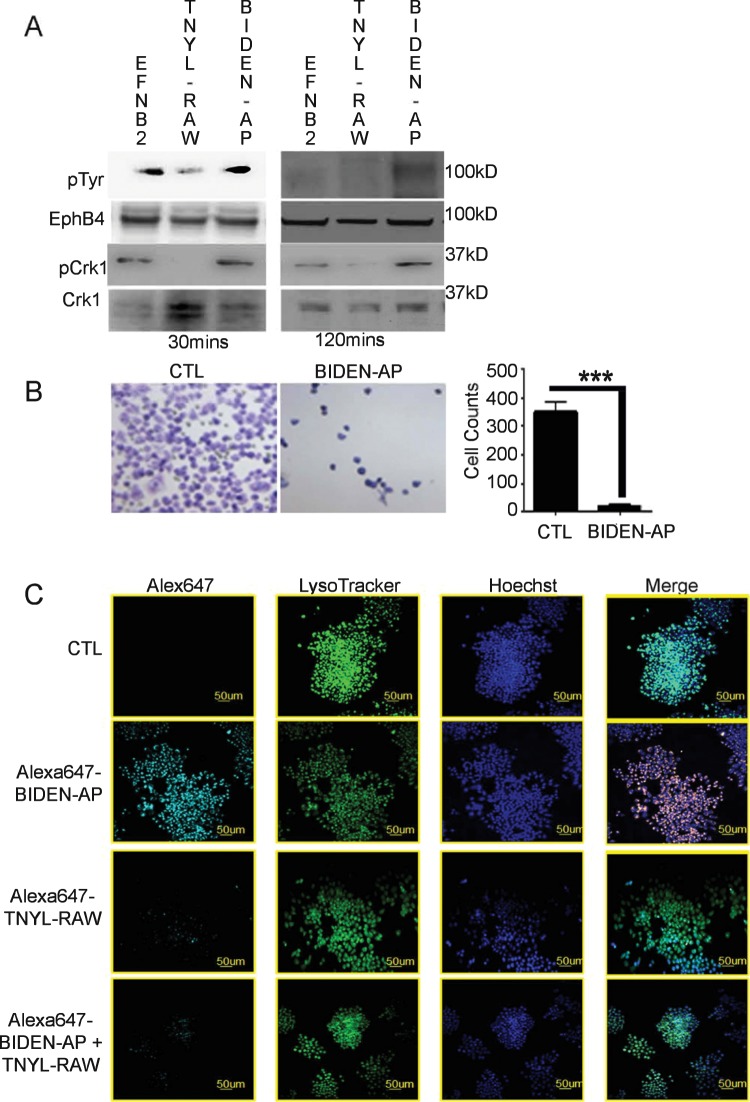
BIDEN-AP acts as an EphB4 agonist. (**A**) BIDEN-AP induced phosphorylation of EphB4 and the associated MAPK14 (Crk1). After 16 h serum starvation, A2780cp20 cells were incubated with EFNB2-Fc (20 nM), TNYL-RAW (50 µM), or BIDEN-AP (50 µM) for 30 min or 2 h. The uncropped blots are presented in Supplemental Figure 11. (**B**) BIDEN-AP inhibited A2780cp20 cell invasion compared to untreated controls (CTL). A modified Boyden transwell chamber coated with Matrigel was used. Representative data from three independent experiments using A2780cp20 cells are presented. Each experiment was repeated in triplicate. Data are shown as mean ± SD. ***p < 0.001 by unpaired, two-sided Student *t*-test. (**C**) Alexa647-BIDEN-AP was internalized via receptor-mediated endocytosis. A2780cp20 cells were incubated at 37 °C with Alexa647-BIDEN-AP (0.05 µM), Alexa647-TNYL-RAW (0.05 µM), or Alexa647-BIDEN-AP (0.05 µM) plus unlabeled TNYL-RAW (5 µM, blocking). Alexa647-BIDEN-AP but not Alexa647-TNYL-RAW was internalized into the A2780cp20 cells.

Next, we determined the phosphorylation of EphB4 and its downstream adaptor protein Crk1 (MAPK14) in lysates from human A2780cp20 ovarian cancer cells by immunoprecipitation with anti-EphB4 and anti-Crk1 antibodies followed by immunoblotting with phospho-specific antibodies. Similar to treatment with EFNB2, BIDEN-AP (but not TNYL-RAW) increased phosphorylation of both EphB4 and Crk1 (Fig. 2A). Functionally, treatment with BIDEN-AP significantly reduced the invasive properties of A2780cp20 cells compared with untreated controls (Fig. 2B), suggesting that BIDEN-AP has a tumor-suppressive property. Alexa647-labeled BIDEN-AP was readily internalized by A2780cp20 cells at 37 °C, as shown by confocal microscopic images, in which Alexa647-labeled BIDEN-AP co-localized with endo-lysosome marker Lysotracker. In comparison, Alexa647-labeled antagonist TNYL-RAW was excluded. Internalization of Alexa647-BIDEN-AP by the A2780cp20 tumor cells was blocked by an excess of unlabeled TNYL-RAW peptide, suggesting that the two peptides were competing for the same EphB4 binding site (Fig. 2C). We also performed the internalization experiment at 4 °C, and no cellular internalization was observed (Supplementary Fig. S4), suggesting that cellular uptake of BIDEN-AP is mediated by energy-dependent endocytosis. Collectively, our data indicate that BIDEN-AP is an EphB4 agonist, activating EphB4 through phosphorylation and receptor internalization.

To analyze the structural basis of the functional switch from an antagonist to an agonist after substitution of a single L-amino acid with its corresponding D-amino acid, we performed computer simulations to model the interaction between BIDEN-AP and EphB4 based on the known crystal structure of the TNYL-RAW/EphB4 complex. Our model suggests that Asn-P2 had minimal interaction with the receptor and that change in configuration of Asn-P2 did not change peptide binding significantly. Change in configuration of L-Tyr-P3 to D-Tyr-P3 resulted in loss of the interaction of the Tyr-P3 side chain with the pocket formed between the D, E, and M strands of the receptor. This allowed the DE loop of the receptor to be more flexible and to move toward agonist conformation to form Van Der Waal interactions with the ligand (Supplementary Data Fig. S5A). Change in configuration of Leu-P4 did not affect the peptide-binding mode, as both L and D conformers occupied the same binding pocket. Significant change in orientation of Phe-P5 was observed when the Phe-P5 configuration was changed from L to D. The side chain of Phe-P5 moved opposite to the Leu95 residue in EphB4 and toward the side chain of Tyr-P3. To accommodate Phe-P5, Tyr-P3 moved slightly away from the pocket but kept the Van Der Waal interactions with the D, E, and M strands, resulting in loss of binding affinity. Change in configuration of Ser-P6 resulted in steric clash with Tyr-P3 and loss of binding affinity. In addition, change in configuration of Pro-P7 and Pro-P10 resulted in a large conformational change in the backbone of the N-terminal residues and C-terminal residues of both TNYL-RAW and BIDEN-AP peptides, which led to the loss of peptide interactions with the receptor. Ile-P11 of the peptide interacted with Leu95 of the receptor. Change in configuration of this amino acid introduced steric clash at this site. This might explain the little to no binding affinity observed after modification with D-Ile-P11. As Trp-P15 is a terminal residue, the side chain has free rotation and binds to the receptor with a similar pose, indicating minimal change in binding affinity as well as activity. These modeling analyses agreed with our detailed SPR binding studies of peptides containing D-amino acids (Supplementary Data Fig. S2**)**.

Our computer modeling revealed that BIDEN-AP had the same orientation as TNYL-RAW to fit into the EphB4 binding cleft (Supplementary Data Fig. S5A). Substitution of L-Tyr-P3 with D-Tyr-P3 reduced steric clash at this site between the BIDEN-AP peptide and the receptor, leading to change in EphB4 conformation, which likely contributed to the switch of the peptide from an antagonistic to an agonistic function. The computational data also support the idea that BIDEN-AP’s binding is specific for EphB4 because the peptide, similar to TNYL-RAW, fitted tightly into the binding cleft of the receptor (Supplementary Data Fig. S5B**)**, while other EphB receptors would have a steric restriction at the Leu95 site (i.e., Arg103 in EphB2 and other EphB receptors)^27^, limiting the fitting of the peptide to their binding pocket. In support of these modeling results, we observed through an ELISA assay that BIDEN-AP bound only to EphB4, but not other B-class Eph receptors (Supplementary Data Fig. S6A**)**. Additionally, we confirmed this binding selectivity using the SPR binding assay (Supplemental Data Fig. S6B).

### Conjugation of BIDEN-AP to core-crosslinked polymeric micelles maintained its binding to EphB4 and agonistic activity

**Figure 3:**
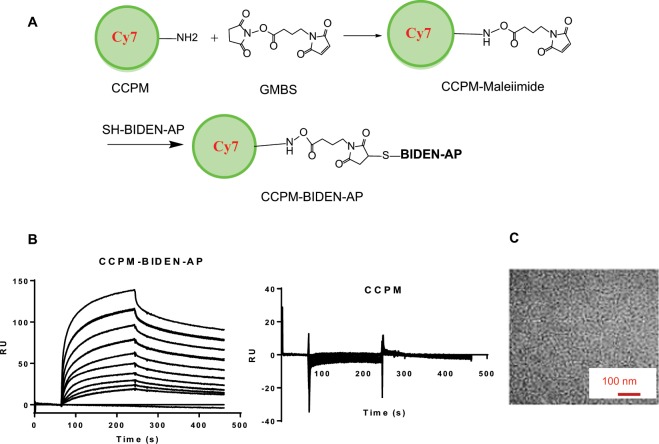
Synthesis and characterization of CCPM-BIDEN-AP nanoconjugate. (**A**) Reaction scheme for the synthesis of CCPM-BIDEN-AP. (**B**) Surface plasmon resonance (SPR) sensorgrams showing high-avidity binding of CCPM-BIDEN-AP to rhEphB4-coated sensor chips. Unconjugated CCPM was used as a control. In both cases, CCPM was used at concentrations corresponding to equivalent BIDEN-AP concentrations in CCPM-BIDEN-AP ranging from 0.16 nM to 20 nM. Duplicates were run for each concentration. RU, response units. (**C**) Transmission electron microscopy of CCPM-BIDEN-AP. The average size of the nanoparticles was 24 ± 3 nm.

Our group previously employed CCPM successfully to improve pharmacokinetics and enhance peptide stability for *in vivo* imaging applications^28–31^. The cross-linked core of CCPM prevents premature micelle disintegration, while the brush-like polyethylene glycol forms a dense protective layer on the micelle surface that minimizes micelle uptake by the organs of the mononuclear phagocytic system. CCPM-TNYL-RAW displayed 4-fold higher systemic exposure than TNYL-RAW (area-under-the time curve [AUC] = 138%ID h/mL versus 29.1%ID h/mL), mainly as a result of significantly slower systemic clearance^30^. We therefore synthesized CCPM-BIDEN-AP using a two-step reaction scheme (Fig. 3A). The resulting conjugate retained the binding affinity to EphB4 (Fig. 3B). We attempted to analyze the binding curves of CCPM-BIDEN-AP to immobilized EphB4 (Fig. 3B) using the conventional (1:1 or 1:2) mass transfer models. However, we could obtain only poor fitting to these models, suggesting that the binding kinetics of CCPM-BIDEN-AP to EphB4 is complex and likely involves multivalency interaction. Through visual inspection of the sensorgrams, we estimated that CCPM-BIDEN-AP displayed stronger binding avidity to EphB4 than the unconjugated BIDEN-AP peptide. The mean diameter of CCPM-BIDEN-AP was determined by transmission electron microscopy to be 24 ± 3 nm (Fig. 3C). Similar to BIDEN-AP, CCPM-BIDEN-AP exhibited potent agonist activity as evidenced by its ability to induce EphB4 and Crk1 phosphorylation *in vitro* in A2780cp20 cells (Supplementary Data Fig. S7A) and *in vivo* in orthotopic A2780cp20 tumors (Supplementary Data Fig. S7B).

### BIDEN-AP–based agents reduced the angiogenic properties of endothelial cells and sensitized endothelial cells resistant to anti-VEGF agents to cell death

**Figure 4:**
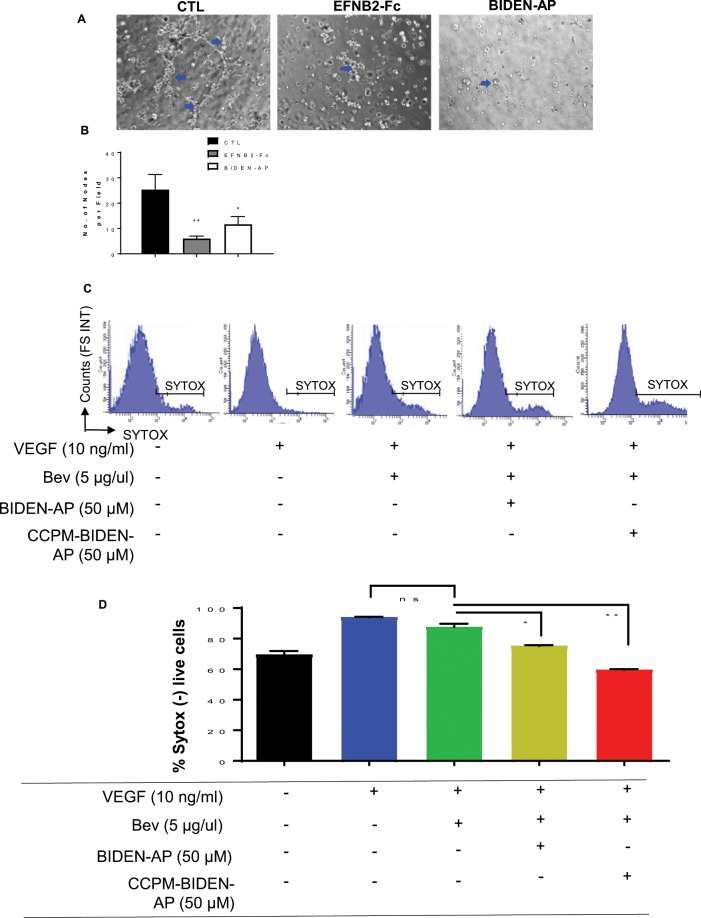
BIDEN-AP–based agents inhibit tube formation and sensitize resistant endothelial cells to anti-VEGF antibody. (**A,B**) Tube formation by RF24 cells treated with EFNB2-Fc (2 nM) or BIDEN-AP (15 µM) compared to untreated controls (CTL). Representative data from three independent experiments are presented. (**A**) Representative microphotographs of tubes formed by RF24 cells are shown (n = 3). Images were taken at × 100 magnification. (**B**) Quantification of the number of nodes per field in three technical replicates. Data are expressed as mean ± SD. *p < 0.05 (CTL vs. BIDEN-AP), **p < 0.01 (CTL vs. EFNB2-Fc) by unpaired, two-sided Student *t*-test. (**C, D**) BIDEN-AP and CCPM-BIDEN-AP induced cell death in bevacizumab-resistant RF24 (R24_Bev) cells. (**C**) Representative flow cytometry plots from three independent experiments of RF24_Bev cells are shown here. SYTOX-negative staining represents the live cell population. (**D**) Percentages of SYTOX-negative live cells from three technical replicates in each experiment are presented as mean ± SD. *p < 0.05 (VEGF + Bev vs. VEGF + Bev + BIDEN-AP), **p < 0.01 (VEGF + Bev vs. VEGF + Bev + CCPM-BIDEN-AP) by unpaired, two-sided Student *t*-test.

We next determined the potential of BIDEN-AP in blocking the reverse EFNB2 signaling in endothelial cells. Our results from a scratch assay using RF24 endothelial cells indicated that BIDEN-AP but not TNYL-RAW (both 50 µM) significantly inhibited cell migration compared to untreated controls (Supplementary Data Fig. S8). Further, both BIDEN-AP and EFNB2-Fc significantly inhibited the formation of capillary-like tubes from RF24 cells (Fig. 4A,B). Therefore, our data showed the potential of BIDEN-AP in compromising the angiogenic property of endothelial cells by interfering with reverse EphB4-to-EFNB2 signaling.

Given the important role of endothelium in adaptive changes with antiangiogenic therapy^32^, we further tested the effects of BIDEN-AP in overcoming resistance to anti-VEGF therapy in RF24 endothelial cells. To do this, we first established a bevacizumab (Bev)-resistant RF24 cell line (RF24_Bev) selected though continuous Bev treatment at low dose (5 µg/µL) for 2 weeks. The RF24_Bev cells were maintained at a Bev concentration of 1.0 µg/µL. Using flow cytometry with SYTOX-green staining, we quantified the live (SYTOX-negative) vs. dead (SYTOX-positive) populations after treatment with BIDEN-AP or CCPM-BIDEN-AP. The results showed that interfering in EphB4-to-EFNB2 signaling with either BIDEN-AP or CCPM-BIDEN-AP re-sensitized these cells to Bev treatment (Fig. 4C,D).

### BIDEN-AP and CCPM-BIDEN-AP displayed potent inhibitory effects on tumor growth and epithelial-mesenchymal transition in an orthotopic human A2789cp20-Luc ovarian cancer xenograft model

**Figure 5:**
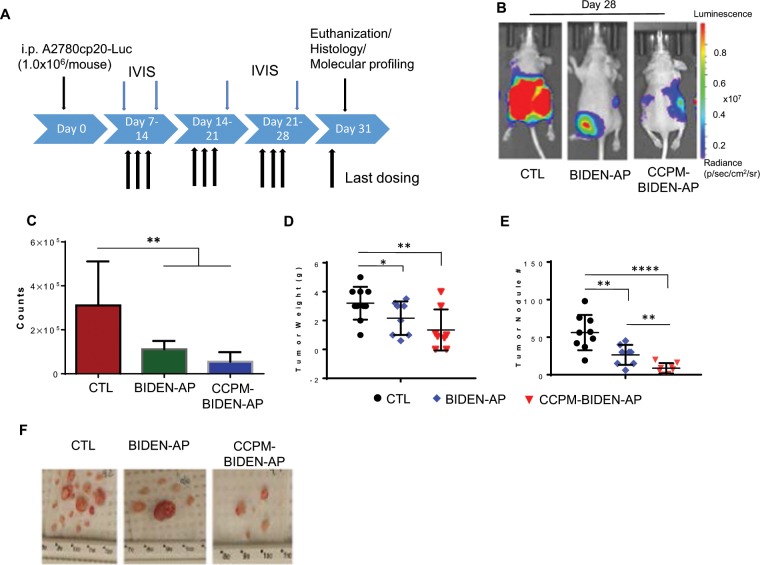
BIDEN-AP and CCPM-BIDEN-AP have potent antitumor activities in orthotopic ovarian cancer model. (**A**) Schema for *in vivo* orthotopic A2789cp20-Luc model and treatment. Female nude mice were intraperitoneally inoculated with A2789cp20-Luc tumor cells. Beginning on day 7 after inoculation, each mouse received 10 intraperitoneal injections of BIDEN-AP or CCPM-BIDEN-AP (bold arrows) at a dose 13 mg/kg/injection every other day for a total of 10 doses. Tumor growth was monitored by bioluminescence imaging (IVIS). (**B,C**) Representative bioluminescence images on day 28 after tumor cell inoculation (**B**) and corresponding quantification of signal intensity (**C**) of mice bearing orthotopic A2780cp20-Luc tumors (n = 4). (**D,E**) Tumor weight **(D)** and number of tumor nodules **(E)** per treatment group (n = 8). (**F**) Images of tumor nodules from each treatment group. Data are expressed as mean ± SD. *p < 0.05, **p < 0.01, ****p < 0.0001 by Student *t*-test. CTL, untreated controls.

**Figure 6:**
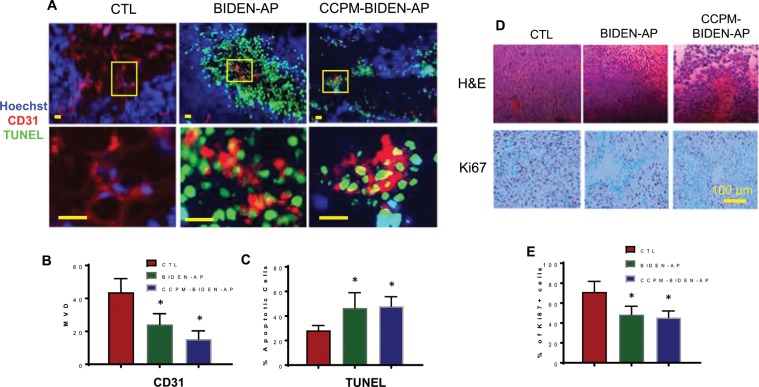
BIDEN-AP and CCPM-BIDEN-AP induce endothelial apoptosis, compromise angiogenesis, and reduce tumor cell proliferation. (**A**) Representative immunofluorescence images of tumor sections stained for CD31 (red) and TUNEL (green). Nuclei were counterstained with Hoechst (blue). Higher magnification images (bottom row) show double-stained apoptotic endothelial cells. Scale bars, 20 µm. (**B, C**) Microvessel density (**B**) and apoptotic (TUNEL-positive) cell counts as a percentage of the total number of cells (**C**). (**D**) Microphotographs of representative hematoxylin and eosin (H&E)– and Ki67-stained sections from each treatment group. (**E**) Ki67-positive cells as a percentage of the total number of cells. Data are derived from 10 high-power fields from three tumors and expressed as mean ± SD. *p < 0.05 (n = 10) compared to untreated control (CTL) by Student *t*-test.

**Figure 7:**
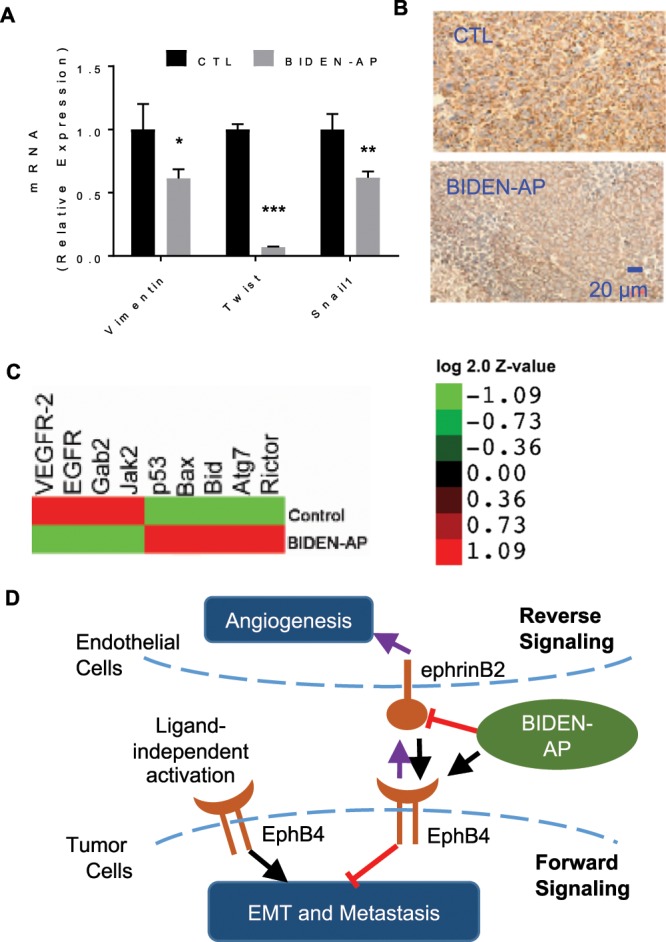
BIDEN-AP–based agents inhibit epithelial-mesenchymal transition signaling. (**A**) qRT-PCR validation of expression of relative mRNA levels of EMT core transcription factors *Twist* and *Snail1* and EMT effector *vimentin* in A2780cp20-Luc tumors from untreated control (CTL) and BIDEN-AP–treated mice. Samples for qRT-PCR were collected from three individual tumors from each group. Data are presented as mean ± SD (n = 3). Significance of differences in results was determined by two-sided Student *t*-test. *p < 0.05, **p < 0.01, ***p < 0.001. (**B**) Microphotographs of representative vimentin-stained tumors from an untreated mouse and a BIDEN-AP–treated mouse. (**C**) Heatmap of selected factors that were most significantly regulated by BIDEN-AP. Data were obtained from reverse-phase protein array of A2780cp20-Luc tumors from untreated mice (control) and BIDEN-AP–treated mice. The median of the expression fold-change from three individual replicas in each condition was plotted in this heatmap as log 2.0 Z-value. (**D**) Proposed mechanism of action of BIDEN-AP. BIDEN-AP–based therapy interferes with bi-directional signaling activities between EphB4 and its membrane-bound ligand EFNB2, which led to suppression of proliferation and metastasis of tumor cells and compromised angiogenesis in surrounding endothelial cells.

We next examined the antitumor activity of BIDEN-AP and CCPM-BIDEN-AP in an orthotopic, luciferase-labeled A2780cp20-Luc ovarian cancer model. Both agents significantly reduced the bioluminescence signal intensity of A2780cp20-Luc on day 28 after initial inoculation (Fig. 5A–C**)**. Both agents significantly reduced tumor growth in the peritoneum compared to controls on the basis of tumor weight and number of metastatic nodules at day 31 after tumor inoculation (Fig. 5D). CCPM-BIDEN-AP reduced metastatic nodules to a greater extent than BIDEN-AP (Fig. 5E,F). Immunofluorescence co-staining using anti-mouse CD31 antibody and TUNEL on the excised A2780cp20 tumors revealed that, in comparison with untreated control tumors, both BIDEN-AP and CCPM-BIDEN-AP induced apoptotic cell death in CD31^+^ endothelial vasculature within tumor stroma; suppressed angiogenesis in tumors as indicated by decreased microvessel density (Fig. 6A–C); and significantly reduced tumor cell proliferation, as evidenced by the decreased number of Ki67-positive cells **(**Fig. 6D,E). As the epithelial-mesenchymal transition (EMT) is characterized by downregulated epithelial cadherin (E-cadherin) expression and upregulated vimentin and neural cadherin (N-cadherin) expression^33^, and results in increased cell invasion, metastasis, and drug resistance^34^, we also determined the key EMT factors from the excised tumors and observed that BIDEN-AP significantly reduced expression of *vimentin*, *Twist*, and *Snail1* at the mRNA level and vimentin at the protein level in A2780cp20 tumors (Fig. 7A,B).

To better understand the mechanism of BIDEN-AP’s action, we compared protein expression profiles of the A2780cp20-Luc tumors harvested from mice that were treated with BIDEN-AP or untreated controls. Integrated pathway analysis of reverse-phase protein array data showed that: 1) regulation of EMT was among the top pathways downregulated by BIDEN-AP, and 2) important regulators involved in tumor-associated angiogenesis, including EGFR, VEGFR2, GAB2, and JAK2, were significantly downregulated by BIDEN-AP, suggesting that the activity of receptor tyrosine kinase–mediated signal transduction pathways EGFR/MAPK14(Crk1)/JAK2 was decreased by BIDEN-AP–based agents. The proapoptotic proteins p53, Bax, and Bid and the autophagy related proteins Atg7 and Rictor were significantly upregulated (Fig. 7C, Supplementary Data Fig. S9 and Table S2), which implicated potential mechanisms for the antitumor effects (Fig. 7D).

### BIDEN-AP displayed antitumor and antiangiogenic effects on a subcutaneous patient-derived xenograft model of ovarian cancer

**Figure 8:**
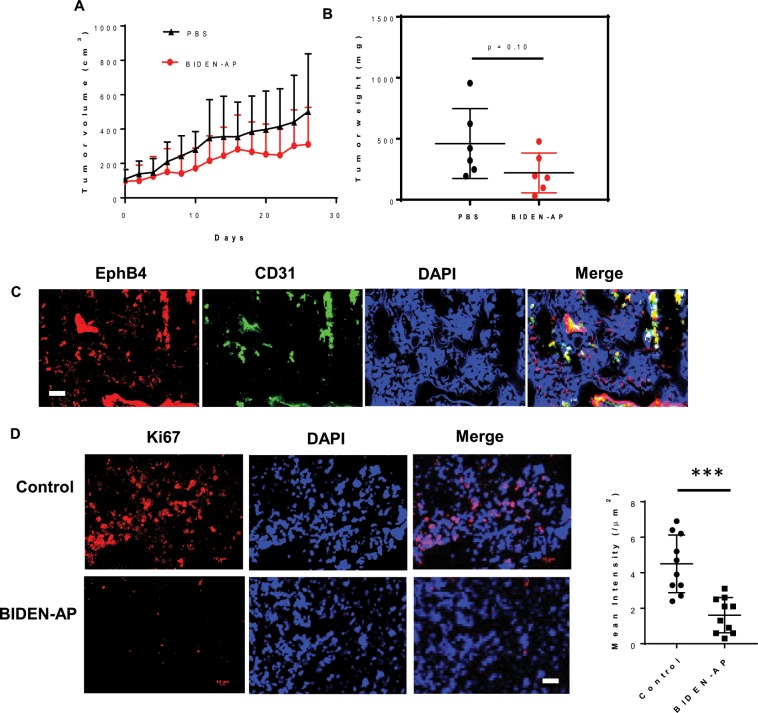
BIDEN-AP–based agents have antitumor activity against an ovarian cancer patient-derived xenograft model. (**A**) Tumor growth curve. (**B**) Scatter plot of tumor weight at the end of a 28-day study. Data are expressed as mean ± SD (n = 6/group). (**C**) Representative immunofluorescence photomicrographs of EphB4- and CD31-stained tumor slices showing EphB4 expression in both tumor cells and tumor vessels. (**D**) Representative immunofluorescence photomicrographs of Ki67-stained tumor slices showing reduced tumor proliferation with BIDEN-AP treatments. The fluorescence intensity of Ki67 staining is expressed as mean ± SD of 10 random fields of view. Magnification of the original slides: ×200. Scale bar, 50 μm. Significance of differences in results was determined by two-sided Student *t*-test. ***p < 0.001.

BIDEN-AP as a single agent delayed the growth of a well-established, subcutaneously implanted MDA-HGSC-1 patient-derived xenograft (PDX) tumor model derived from a patient with high-grade serous carcinoma (HGSC) (Fig. 8A). At the end of the study (28 days after initiation of treatments), the tumor weights were 461 ± 286 mg^3^ and 220 ± 164 mg^3^ for mice treated with phosphate-buffered saline solution (PBS) and those treated with BIDEN-AP, respectively (p = 0.10, Student *t*-test) (Fig. 8B; Supplementary Data Fig. S10A). However, BIDEN-AP did not cause body weight change compared to mice in the control group (Supplementary Data Fig. S10B). Immunofluorescence co-localization analysis showed that EphB4 was expressed in both tumor vasculature and tumor cells in the MDA-HGSC-1 PDX model (Fig. 8C). Tumor cell proliferation was significantly reduced (p < 0.001) by BIDEN-AP treatments (Fig. 8D). Thus, BIDEN-AP showed therapeutic potential as a monotherapy against a clinically relevant HGSC-PDX tumor.

## Discussion

The dual function of EphB4 as tumor promoter or suppressor associated with its EFNB2 ligand opens a therapeutic window for modulating EphB4 activity in tumor cells^14^. While exploring the effects of structural modifications to the binding affinity of EphB4 antagonist TNYL-RAW peptide^21,22^, we unexpectedly found that substitution of L-Tyr-P3 with D-Tyr-P3 led to a functional switch from EphB4 antagonist to EphB4 agonist. Unlike EphB4′s natural ligand EFNB2, which binds to several receptors within the B subclass^23,26^, our EphB4 agonist peptide, BIDEN-AP, exclusively bound to EphB4. Further experiments indicated that both BIDEN-AP and its nanoconjugate CCPM-BIDEN-AP promoted forward, tumor-suppressive EphB4 signaling both *in vitro* and *in vivo*; blocked the reverse signaling by interfering in the interaction between EphB4 and its natural ligand EFNB2 in endothelial cells, thereby suppressing these cells’ angiogenic properties; and sensitized Bev-resistant endothelial cells to cell death. Furthermore, monotherapy with BIDEN-AP or CCPM-BIDEN-AP demonstrated significant antitumor effects and antiangiogenic activity in an orthotopic human ovarian cancer model and was active against tumor growth in an HGSC-PDX model.

Our functional studies showed that internalization of BIDEN-AP was blocked by a large excess of TNYL-RAW peptide, suggesting that both peptides compete for the same EphB4 binding pocket. Further study showed that BIDEN-AP also competed with EFNB2-Fc for binding to EphB4. Given that X-ray crystallography studies have shown that TNYL-RAW and the receptor binding site of EFNB2 fit into the same binding pocket in EphB4^23,27^, we conclude that BIDEN-AP, like TNYL-RAW, also fits into the same binding pocket in EphB4.

Using the X-ray structure of TNYL-RAW bound to human EphB4 as a structural guide^23^, we generated binding modes for BIDEN-AP in the binding pocket of EphB4 for both EFNB2 and TNYL-RAW^23,27^. In comparing EphB4-TNYL-RAW and EphB4-BIDEN-AP, we found significant deviation throughout the structure of the receptor loop regions. Dynamic modeling of the binding mode of BIDEN-AP revealed a hydrogen-bonding network that closely resembled that observed in the EphB4-EFNB2 crystal structure. On the basis of these computational modeling studies, we believe that the loss of interaction of the Tyr-P3 side chain with the D, E, and M strands of the receptor might be the mechanism for this drastic change in functional activity. The computational model also supported the idea that change in conformation of the DE loop might be involved in determining the activity of the ligand and the response of the receptor^27^. These results highlight how structural modifications may affect functional activity, as substitution of a single amino acid from an L- to a D-form resulted in transformation to a 15-mer peptide that had a completely different functional response.

N-terminal Tyr-P3 of the antagonist peptide is reportedly required for high-affinity binding as well as for efficient antagonistic properties, whereas residues such as Leu-P4, Phe-P5, and C-terminal residues Ile-P11 and Trp-P15 form favorable interactions with Leu95 of the EphB4 receptor and play important roles in specificity^23^. The interactions of the peptides with Leu95 of the receptor are unique to EphB4. The corresponding strictly conserved residue Arg-103 in other members of the EphB receptor subclass would form steric clashes with ligand atoms. Therefore, our computational data aligned consistently with our experimental conclusion that BIDEN-AP bound selectively to EphB4.

Previous work reported the use of monoclonal antibodies, soluble fusion proteins, and small-molecule kinase inhibitors to inhibit the reverse EphB4-to-EFNB2 signaling, which promotes angiogenesis^20,35–37^. At present, a drug targeting EphB4-EFNB2 protein-protein interaction, based on the recombinant soluble EphB4–human serum albumin fusion protein (sEphB4-HSA), has advanced to phase I clinical testing (NCT02495896)^12,38^. sEphB4-HAS inhibited the activity of VEGF^39^ and does not activate forward EphB4 signaling. Previous knowledge suggested that, in the absence of functional forward signaling, a ligand-independent mechanism may induce tumorigenesis in tumors with a high level of EphB4 expression^40,41^. For example, a kinase-deficient EphB4 mutant was still capable of increasing breast cancer cell growth^42^. Collectively, BIDEN-AP and its nanoconjugate CCPM-BIDEN-AP represent a new class of agents that can effectively suppress tumor growth by promoting ligand-dependent forward EphB4 signaling in tumor cells and reduce angiogenesis by inhibiting reverse EFNB2 signaling in tumor-associated endothelial cells (Fig. 7D).

In summary, our studies provide comprehensive evidence to support the use of BIDEN-AP for targeting bi-directional EphB4/EFNB2 signaling to reduce tumor growth and metastasis and to overcome resistance to antiangiogenic therapy. These results show the preclinical potential for modulating the EphB4/EFNB2 protein interfaces and their mode of interaction, which will facilitate further development of therapeutic agents.

## Materials and Methods

### Cell lines

All cells were maintained in 5% CO_2_, 95% air at 37 °C. Ovarian cancer HeyA8 and cisplatin-resistant A2780Cp20 cells were maintained in Roswell Park Memorial Institute (RPMI) 1640 medium with 15% heat-inactivated fetal bovine serum (FBS) plus 0.5% gentamicin as described previously^43,44^. All cell lines were routinely tested to confirm the absence of mycoplasma (Gen-Probe detection kit; Thermo Fisher Scientific, Carlsbad, CA) and were validated by the MD Anderson Cancer Center Characterized Cell Line Core Facility. All *in vitro* experiments were conducted with 60–80% confluent cultures.

RF24 endothelial cells were a kind gift of Arjan W. Griffioen (Maastricht University Hospital, Maastricht, The Netherlands) and were validated by short tandem repeat profiling^45^. RF24 cells were maintained in modified essential medium (MEM) supplemented with 10% FBS, sodium pyruvate, MEM vitamins, L-glutamine, and MEM non-essential amino acids.

### Protein-based binding assay to EphB class receptors

The surface plasmon resonance (SPR) technique was used to investigate binding kinetics, competition, and binding selectivity to EphB4 using the BIACore 3000 system (GE Healthcare Life Sciences, Pittsburgh, PA). Detailed description of the SPR assay is provided in the Supplementary Information.

### ELISA assay for testing the binding selectivity

Briefly, biotinylated BIDEN-AP was immobilized on streptavidin-coated 96-well plates (Pierce, Waltham, MA) at a concentration of 10 µM. After washings, wells were incubated with Eph ectodomain proteins EphB1, EphB2, EphB3, EphB4, or EphB6 fused with Fc (2.5 µg/mL) in binding buffer (0.5% bovine serum albumin [BSA] in PBS) at room temperature for 1 h. The plates were washed again with binding buffer, and bound receptors were detected using an anti-Fc antibody coupled to alkaline phosphatase (Promega, Madison, WI), followed by addition of *p*-nitrophenyl phosphate as the substrate (Pierce). The signals were measured at 405 nm. Alkaline phosphatase activity from wells without EphB-Fc was subtracted as background. All signals were normalized to the value measured with EphB4-Fc.

### Cell-based binding assay

A2780cp20 ovarian cancer cells (1×10^6^ cells/mL) were incubated with EFNB2-Fc (20 nM) and BIDEN-AP at concentrations ranging from 0 to 100 µM at 25 °C for 1 h. After washings, EFNB2-Fc bound to the cells were probed with phycoerythrin (PE)-labeled anti-Fc antibody and analyzed by flow cytometry. Binding of BIDEN-AP to A2780cp20 cells was determined by its capacity to displace EFNB2-Fc’s binding to the tumor cells, measured by decreased PE fluorescence signal. Cells were analyzed in a Cellquest fluorocytometer (BD Bioscience, San Jose, CA). The experiments were run in duplicate.

### Modeling BIDEN-AP-EphB4 binding

The crystal structure of the peptide antagonist (TNYL-RAW) bound to EphB4 (Protein Data Bank ID: 2BBA) (10.1016/j.str.2005.11.011) was imported into the Molecular Operating Environment (MOE) 2016 software (Chemical Computing Group, Montreal, Canada). Structure preparation wizard was used with default settings to add missing atoms and partial charges. Energy was minimized by using default settings. The configuration of each amino acid of the antagonist peptide was changed from L to D serially using MOE’s Protein Builder tool. The resulting complexes were energy-minimized using default settings.

### Invasion assays

The membrane invasion culture system chamber, a modified Boyden transwell chamber, was used to measure the invasive potential of all cell lines used in this study. A2780cp20 tumor cells (7.5 × 10^4^) treated with BIDEN-AP or TNYL-RAW or untreated were suspended in 100 µL serum-free medium and were added into the upper chambers, which were pre-coated with human defined matrix composed of 50 µg/mL human laminin (Sigma L6274), 50 µg/mL human type IV collagen (Sigma C6745) in 10 mM acetic acid, and 2 mg/mL gelatin (Sigma G1393)^46^. Complete medium containing 10% FBS (500 µL) was added to the bottom chambers as a chemoattractant. The chambers were incubated at 37 °C in 5% CO_2_ for 24 h. After incubation, the cells on the inner side of the upper chambers were removed with cotton swabs. The cells on the outside of the upper chambers, the invaded cells, were fixed, stained, and counted by light microscopy. Quantification of invaded cells were performed using Image J 1.8.0_172 plugin^47^. Cells from five random fields per treatment were counted.

### Endothelial cell migration assay

RF24 cells were seeded in a 6-well plate (Corning Inc., Corning, NY), 2.5 × 10^5^ cells/well in 2.5 mL modified essential medium (MEM) supplemented with 10% FBS. Cells were serum-starved for 24 h when they reached about 90% confluency. The cell monolayer was scratched with a sterile pipet tip to make a “wound.” The growth medium was then removed and the cell layer was washed three times with serum-free medium to remove the detached cells. MEM medium (0.5% FBS) alone (control) or the medium plus either TNYL-RAW or BIDEN-AP at a concentration of 50 µM was added to each prepared well, and the cells were incubated. The width of the scratch was documented by microphotograph at 6 h. The percentage of the wounded area was measured and calculated by using ImageJ Version 1.51 (National Institutes of Health, Bethesda, MD). Experiments were performed in seven replicates. The data are expressed as mean ± standard deviation (SD).

### Assay of tubule formation of immortalized endothelial cells

Matrigel (Becton Dickinson, Bedford, MA) was used to assess formation of capillary-like structures by RF24 cells on a basement membrane. Twelve-well Costar plates (Corning, Corning, NY) were coated with Matrigel (10 mg/mL) according to the manufacturer’s instructions. RF24 cells (1.0 × 10^4^ cells/well) were seeded on Matrigel-coated plates and incubated at 37 °C for 60 min. EFNB2-Fc (2 nM) or BIDEN-AP (15 µM) was added, and the cultures were incubated at 37 °C for 24 h. *In vitro* endothelial tubule formation was observed and photographed after 24 h as described previously^46^. The degree of tubule formation was determined by the number of nodes. The node was defined at least 3 or more cells formed a single jointed point. The nodes for each image were counted manually. Five images (×100 magnification) per treatment were counted double blindly. Data are expressed as a percentage of the number of tubes in untreated control wells.

### Orthotopic ovarian cancer models

All mouse studies were approved by the Institutional Animal Care and Use Committee (IACUC) at The University of Texas MD Anderson Cancer Center (IACUC protocol 00001332). Mice were cared for in accordance with guidelines set forth by the American Association for Accreditation of Laboratory Animal Care and the US Public Health Service Policy on Human Care and Use of Laboratory Animals. Female athymic nude mice (NCr-nu) were obtained from Harlan Laboratories (Indianapolis, IN) at age 4 to 6 weeks and kept under sterile conditions. For all therapeutic experiments, groups of the mice were inoculated intraperitoneally with A2780cp20-Luc cells (1×10^6^ cells/mouse). The mice were treated with BIDEN-AP or CCPM-BIDEN-AP at a dose of 13 mg equivalent drug/kg per injection for a total of 10 doses every other day starting on day 7 after tumor cell inoculation. Untreated mice were used as a control. On day 31, all mice were euthanized and necropsied, and their tumors were harvested. Tumor weight and number and location of tumor nodules were recorded. Tumor tissue was fixed in formalin for paraffin embedding, frozen in optimal cutting temperature medium for frozen slide preparation, or snap-frozen for lysate preparation.

### PDX model of high-grade serous ovarian carcinoma

Under Institutional Review Board (IRB protocol PA15-0441) and IACUC approval at MD Anderson Cancer Center, patients with HGSC who were being treated at MD Anderson gave consent for the use of their tumor tissue in this study. Four- to six-week-old female Balb C athymic nude mice (Harlan Laboratories) were implanted with one PDX 3×3×3 mm tumor cube (MDA-HGSOC-1 high-grade serous ovarian cancer; generation 9) in the dorsal flank to create subcutaneous HGSC-PDX model. Tumor size was monitored by caliber, and tumor volume was calculated using the formula V(cm^3^) = (length x width^2^)/2. Mice were randomly assigned to the PBS vehicle control or BIDEN-AP treatment group, 6 mice per group, once tumor size reached ~100 mm^3^ (21 days after implantation). Treatments consisted of intraperitoneal injection of PBS or BIDEN-AP at 300 μg/injection (100 μL/injection) once every other day for a total of 14 doses. At the end of the study (day 49), all mice were euthanized, and tumors were collected, weighed, snap-frozen in optimal cutting temperature compound with liquid nitrogen, and cryosectioned into 5-μm sections for immunohistochemical staining of CD31 and Ki67 markers.

### Bioluminescence imaging

On day 7, day 14, day 21, and day 28 after tumor inoculation, mice received a tail vein injection of D-luciferin (4 mg/kg) and their tumor burden was assessed by luciferase activity using the Xenogen IVIS-200 imaging system (Perkin Elmer, Shelton, CT). The bioluminescence imaging signal generated in mice was quantified using Living Image V.2.11 software and IGOR image analysis software (V.4.02 A; WaveMetrics, Portland, OR). The region of interest was manually selected, and the signal intensity was expressed in terms of the number of photons/s/cm^2^.

### Statistical analysis

In general, all *in vitro* experiments, including cell proliferation, viability, migration, and invasion, unless stated otherwise, were done in three, six, or seven replicates. For each *in vitro* experiment dataset (Figs. 2, 4 and 7, Supplementary Fig. S8), a two-tailed Student *t*-test (for comparisons of two groups) and analysis of variance (for comparisons of more than two groups) were applied to determine the significance of differences in results. For each *in vivo* experiment, the tumor weight and number of metastatic nodules were analyzed by one-way analysis of variance, and groups were compared by independent sample *t*-test. The SPSS statistical software (IBM, Armonk, NY) was used for all analyses (Figs. 5, 6 and 8). All data are presented as mean ± SD unless otherwise indicated. Differences were considered statistically significant if *p* < 0.05.

### Statement of significance

Preclinical evidence presented here shows that peptide-based BIDEN-AP agents promoted tumor-suppressive, EphB4-mediated forward signaling in ovarian cancer cells and simultaneously inhibited tumor-promoting, EFNB2-mediated reverse signaling in endothelial cells. These data have direct relevance for developing BIDEN-AP–based agents as a new therapeutic approach for treatment of EphB4-positive ovarian cancer.

## Supplementary information

Supplemental Information.
