# Supplementary material for: Targeting Forward and Reverse EphB4/EFNB2 Signaling by a Peptide with Dual Functions
Source: Sci Rep. 2020 Jan 16;10:520. doi: 10.1038/s41598-020-57477-x (PMC6965176; doi:10.1038/s41598-020-57477-x)
Supplement: Supplementary file 1 — Supplemental Information. [file 41598_2020_57477_MOESM1_ESM.pdf]

Supplemental Materials

Targeting Forward and Reverse EphB4/EFNB2 Signaling by a Peptide with Dual Functions

Running Title: Targeting EphB4/EFNB2 bi-directional signaling

Chiyi Xiong[1†], Yunfei Wen[2†], Jun Zhao[1], Dengke Yin[1,3], Lingyun Xu[1,4], Anca Chelariu-Raicu[2], Cody Yao[1], Xiaohong Leng[5], Jinsong Liu[5], Rajan R. Chaudhari[6], Shuxing Zhang[6], Anil K. Sood[2,7], and Chun Li[1,7]*

Supplemental materials and methods, 2 Supplementary Tables and 11 Supplemental Figures are available in the online version of the paper.

Supplemental Materials and Methods

Materials All amino acid derivatives and coupling reagents were purchased from Novabiochem (San Diego, CA), Bachem (Torrance, CA), or Chem-Impex International (Wood Dale, IL). All other chemicals were purchased from Sigma-Aldrich (St. Louis, MO). Reagent-grade solvents were used without further purification unless otherwise specified. Recombinant EphB4/Fc chimera, phycoerythrin (PE)-conjugated rat anti-human EphB4 monoclonal antibody, and rabbit anti-EphB4 antibody were purchased from Santa Cruz Biotechnology (Santa Cruz, CA). The BIACore sensor chip CM5, amine coupling kit, HBSEP running buffer (0.01 M [4-(2-hydroxyethyl)-1-piperazineethanesulfonic acid] [HEPES], pH 7.4, 0.15 M NaCl, 3 mM ethylenediaminetetraacetic acid [EDTA], and 0.005% [v/v] surfactant P20 solution), and regeneration buffer were purchased from BIACore, Inc. (Piscataway, NJ). Human EphB4 and murine EphB4, EphB1, EphB2, EphB3, and EphB6 were purchased from R&D Systems (Minneapolis, MN). Phycoerythrin-labeled anti-Fc antibody and EFNB2-Fc were obtained from Sino Biological (Beijing, China).

Peptide synthesis. Solid phase syntheses were carried out on the automatic peptide synthesizer Prelude (PTI, Tucson, AZ) using Rink resin (Novabiochem). The resin (0.05-0.1 g) was swollen and washed with 5×1.5 mL of dimethylformamide (DMF)/methylene chloride (CH_2Cl_2). Fluorenylmethyloxycarbonyl (Fmoc) groups were removed with 3×1.5 mL of 20% piperidine/DMF for 5 min each. For coupling, three-fold excesses of Fmoc-amino acids, diisopropylcarbodiimide, and 1-hydroxybenzotriazole in 3 mL of DMF/CH_2Cl_2 were used. This procedure was repeated once. After coupling and deprotection steps, resins were washed with 3×3 mL of DMF/CH_2Cl_2. On completion of the peptide chain elongation, resins were washed with 3×3 mL of CH_2Cl_2 and were treated with trifluoracetic acid : triisopropylsilane : H_2O (95:2.5:2.5) for 15 min each. The combined filtrates sat at room temperature for 1-2 h, and the volumes were reduced in a vacuum. Peptides were precipitated in ice cold ethyl ether, collected by centrifugation, washed two times

with ethyl ether, and subjected to centrifugation. After drying, peptides were purified by reverse-phase high-performance liquid chromatography (HPLC) on an Agilent 1200 system (C-18, Vydac, 10×250 mm, 10 µm; Santa Clara, CA). Alexa647-coupled TNYL-RAW or BIDEN-AP were obtained by solution phase reaction of the respective peptide with Alexa647-N-hydroxysuccinate (Alexa647-NHS), followed by HPLC purification.

CCPM-BIDEN-AP conjugation. CCPM with amine functionalized surface was synthesized according to reported procedures (*21*). To introduce a maleimide group to the CCPM, an aliquot of N-(γ-maleimidobutyryloxy)-succinimide ester) (GMBS) in DMF (22.5 mL, 7 mmol/mL, 0.16 mmol) was added into the CCPM (250 mL, 2.6 nmol nanoparticles) in 1.2 mL of phosphate-buffered saline solution (PBS, pH 8). The mixture was stirred for 3 h at 37ºC. The product was purified using a PD-10 column to remove unreacted GMBS. To introduce a sulfhydryl group to BIDEN-AP, the amino acid cysteine was conjugated to the N-terminal amine of BIDEN-AP. The resulting sulfhydryl-containing Cys-BIDEN-AP was mixed with 2 mL of a solution of CCPM-maleimide (0.16 mmol equivalent maleimide) in PBS with a 2:1 molar ratio of Cys-BIDEN-AP to maleimide groups (CCPM). The solution was stirred for 12 h at 4ºC and then purified using a fast protein liquid chromatography system (Amersham Pharmacia Biotech, Uppsala, Sweden) equipped with a G200 column and an ultraviolet light detector (254 nm). The column was eluted with PBS to remove unreacted Cys-BIDEN-AP. The unreacted Cys-BIDEN-AP was quantified on an Agilent 1100 Series LC/MSD-TOF instrument equipped with a Vydac C18 column. The molar ratio of BIDEN-AP to CCPM was calculated on the basis of Cys-BIDEN-AP consumed (total Cys-BIDEN-AP minus remaining Cys-BIDEN-AP). The size of CCPM-BIDEN-AP was determined by transmission electron microscopy (TEM). Briefly, a drop of aqueous sample solution was placed on a 400-mesh copper grid coated with 0.5% poly(vinyl formal) aqueous solution (w/w). A droplet of 1% uranyl acetate solution was added for negative staining. The sample was air-dried and

examined with a JEM 1010 transmission electron microscope (JEOL USA, Inc., Peabody, MA) at an accelerating voltage of 80 kV. Digital images were obtained using the AMT Imaging System (Advanced Microscopy Techniques Corp., Danvers, MA).

Surface plasmon resonance (SPR) binding and competition assays. A stock solution of EphB4 in PBS (100 µg/mL) was diluted to 25 µg/mL with 10 mM sodium acetate buffer (pH 4.5) and immobilized to CM5 sensor chips using the amine coupling reaction according to manufacturer-provided procedures (BIACore). Briefly, the surfaces of the chips in flow cells (FC)-1 and FC-2 were activated by exposing chips to a mixture of 200 mM N-ethyl-N'-dimethylaminopropyl carbodiimide and 50 mM N-hydroxysuccinimide for 7 min. FC-1 was used as a reference surface and was directly deactivated by injecting 1 M ethanolamine at pH 8.5 for 7 min. FC-2 was injected with 25 µg/mL human or murine EphB4 followed by injection of 1 M ethanolamine to block the remaining activated ester groups on the surface. The chips were allowed to stabilize for at least 2 h in HBSEP running buffer before injection of test analytes.

Binding assays were performed in duplicate at 25°C in HBSEP running buffer. Test peptides were diluted in HBSEP buffer, filtered, degassed, and injected at serially doubled concentrations, from 1.6 nM to 800 nM, at a flow rate of 30 µL/min. The injection time of test peptides into the HBSEP buffer was 7 min, followed by a 3-min dissociation period. The chips were regenerated using a 30-s pulse of 10 mM glycine (pH 2.2) after each binding circle. Each cycle consisted of a 2-min waiting period to allow monitoring of the baseline binding stability. A double-referencing procedure was performed to subtract bulk effects caused by changes in the buffer composition or nonspecific binding. Thus, all analyzed samples, including a sample of the running buffer, were additionally injected onto an uncoated reference surface. The association rate (K_{on}) and dissociation rate (K_{off}) were calculated by fitting binding chromatogram data using the BIACore evaluation software with a 1:1 Langmuir binding model. The binding constant K_D was

calculated as K_{off}/K_{on}. Buffer alone and EFNB2 were used as negative and positive controls, respectively.

The capacity of BIDEN-AP to inhibit the binding of EFNB2 to EphB4 was assessed in a competitive binding assay. A stock solution of EFNB2-Fc in PBS (100 µg/mL) was diluted to 25 µg/mL with 10 mM sodium acetate buffer (pH 4.5) and was immobilized to CM5 sensor chips as already described. Serial dilutions of BIDEN-AP ranging from 2 to 1000 nM were mixed with human EphB4 (30 nM) and injected onto EFNB2-Fc–coated CM5 chips. After each injection, the signal from the control flow cell was subtracted and the relative amount of EphB4 bound to EFNB2-Fc was recorded as the net response over the pre-injection baseline level. A double-referencing procedure was performed to subtract bulk effects caused by changes in the buffer composition or nonspecific binding.

Immunoblotting, immunoprecipitation, and co-immunoprecipitation. For immunoblotting, lysates from cultured cells were prepared using modified radioimmunoprecipitation assay (RIPA) buffer (50 mM Tris–HCl [pH 7.4], 150 mM NaCl, 1% Triton, 0.5% deoxycholate) plus 25 µg/mL leupeptin, 10 µg/mL aprotinin, 2 mM EDTA, and 1 mM sodium orthovanadate. To prepare lysates of snap-frozen tissue from mice, approximately 30-mm^3 cuts of tissue were disrupted with a tissue homogenizer and subjected to centrifugation at 13,000 rpm for 30 min in modified RIPA buffer. The protein concentrations were determined by using a BCA Protein Assay Reagent kit (Pierce Biotechnology, Rockford, IL). Lysates were loaded and separated by 8% sodium dodecyl sulfate (SDS)—polyacrylamide gel electrophoresis (PAGE). Proteins were transferred to a nitrocellulose membrane by semidry electrophoresis (Bio-Rad Laboratories, Hercules, CA) overnight, blocked with 3% BSA for 1 h, and then incubated at 4°C with primary antibody overnight. After washing with a mixture of tris-buffered saline and Tween 20 (TBST) solutions, the membranes were incubated with horseradish peroxidase (HRP)-conjugated horse anti-mouse IgG (1:2000, GE Healthcare, Amersham Place, UK) for 2 h. HRP was visualized by an enhanced

chemoluminescence detection kit (Pierce). To confirm equal sample loading, the blots were probed with an antibody specific for beta-actin (0.1 µg/mL; Sigma-Aldrich). Densitometry was performed using ImageJ software. For immunoprecipitation and co-immunoprecipitation, cells were subjected to lysis in non-denaturing NP40 cell lysis buffer (Cell Signaling Technology, Boston, MA). The extracts were incubated with A/G-conjugated antibody at 4°C for 2 h, and for immunoprecipitation the beads were washed twice with RIPA buffer, once with 0.5 m LiCl in 0.1 m Tris (pH 8.0), and once with PBS. Reactions were boiled in sample buffer, and proteins were then subjected to 10% SDS-PAGE and immunoblotting.

Immunofluorescence imaging. A2780cp20 ovarian cancer cells (5×10^5 cells/well) were plated in 4-well chamber slides and treated with Alexa647-labeled BIDEN-AP (50 µM) for 1 h or 2 h. Cells were washed three times with 1× PBS and fixed in freshly prepared fixative containing 3.7% formaldehyde, 0.05% glutaraldehyde, and 0.4% Triton-X-100 in PHEMO buffer (0.068 M PIPES, 0.025 M HEPES, 0.015 M EGTA, 0.003 M $MgCl_2$, and 10%v/v dimethylsufoxide [DMSO]) for 10 min at room temperature. LysoTracker Green (Cat. L7526; Invitrogen, Carlsbad, CA) was used as a marker of lysosome. The nucleus was stained with Hoechst 33342 (300 nM) at room temperature for 10 min before mounting in Gel Mount mounting medium (Vector Laboratories, Burlingame, CA). Fixed and immunofluorescence-stained cells were imaged using a Zeiss LSM510 Meta laser scanning confocal system (Zeiss, Dublin, CA) configured to a Zeiss Axioplan 2 upright microscope with a CFI Plan Apo Lambda *20×* objective lens. Images were obtained from three independent biological replicates.

Immunofluorescence analysis of CD31 was performed on 5-µm-thick fresh-frozen tissue samples. After deblocking with acetone, acetone-chloroform (1:1), and acetone for 5 min, non-specific binding was blocked with 4% fish gelatin (Biotium Inc, Hayward, CA) in PBS for 30 min. The primary antibody, rabbit polyclonal anti-mouse CD31 (ab28364; Abcam, Cambridge, MA) at

1:500 dilution (100-200 µL) in 4% fish gelatin was applied to each slide and the slides were incubated overnight at 4°C. After washing, the slides were incubated with Alexa488-conjugated goat anti-rabbit secondary antibody for 60 min. Slides were subjected to recombinant terminal deoxynucleotidyl transferase (rTdT)–mediated dUTP nick-end labeling (TUNEL) by using the DeadEnd Fluorometric TUNEL System assay kit (Promega) after CD31 staining by immersing slides in 4% formaldehyde in PBS for 15 min. A 20 µg/mL proteinase K solution (Promega) was added to each slide (100 µL per slide), and the slides were incubated at room temperature for 8-10 min. Slides were washed twice with PBS, 100 µL equilibration buffer (Promega) was added to each, and the slides were again incubated for 5-10 min. The slides were then labeled by adding 50 µL of fluorescein-12-dUTP as a substrate, and the rTdT was added. Each slide was covered with a plastic coverslip to ensure even distribution of the labeling solution and incubated for 60 min at 37°C in a humidified chamber. The reaction was stopped by immersing slides in 2x SSC solution (87.7g NaCl, 44.1g sodium citrate, pH 7.0, in 500 mL) for 15 min. After washing with PBS, the slides were counterstained with Hoechst (Sigma-Aldrich) for 15 s. To acquire immunofluorescence images of tissue slices, the slides were mounted and viewed under a Zeiss Axiovert Z.1 fluorescent microscope (Zeiss, Jena, Germany). Microvessel density was evaluated by calculating the average number of CD31-positive microvessels in ten randomly selected tumor areas from each of three tumor samples at x200 magnification. The apoptotic index was calculated as TUNEL-positive cells/total number of cells in ten randomly selected areas at x200 magnification from three tumor samples.

Immunofluorescence staining of the samples from resulting PDX tumors was done similarly. For EphB4 staining, slides were probed using rabbit anti-human EphB4 polyclonal antibody (Abcam ,Cambridge, MA) as the primary antibody and Alexa Fluor 594-conjugated goat anti-rabbit immunoglobulin (Abcam) as the secondary antibody. For CD31 staining, slides were stained with rat anti-mouse CD31 antibody (Clone 390, Biolegend, San Diego, CA) and Alexa Fluor 488-conjugated donkey anti-rat antibody (Abcam). Ki67 was stained with rabbit anti-

Ki67antibody (Clone SP6, Cell Marque, Rocklin, CA) and Alexa Fluor 594-conjugated goat anti-rabbit antibody (Abcam). Cell nuclei were counterstained with DAPI.

Immunohistochemistry (IHC) analysis. For IHC analysis of Ki67, formalin-fixed, paraffin-embedded tumor sections were deparaffinized and rehydrated. After antigen retrieval, slides were blocked in PBS with 10% goat serum and incubated with primary antibody anti-Ki67 (1:400 dilution; Abcam) overnight at 4°C. Slides were washed and incubated with biotinylated goat-anti-rabbit IgG (1:200; Vector Laboratories) and streptavidin-conjugated HRP (DAKO, Carpinteria, CA) for 30 min each. A positive reaction was detected by exposure to 3,3′-diaminobenzidine. Slides were counterstained with hematoxylin and visualized under a microscope. The positively stained nuclei were counted in at least ten randomly selected 20x fields of view.

Integrated pathway analysis of reverse-phase protein array. Three individual tumors with comparable sizes from each group were subjected to prepare for protein lysate. Briefly, the small piece of tumor tissue was put into a 5 ml tube on ice, and added ice-cold lysis buffer (1% Triton X-100, 50 mM HEPES, pH 7.4, 150 mM NaCl, 1. 5mM $MgCl_2$, 1 mM EGTA, 100 mM NaF, 10 mM Na pyrophosphate, 1 mM Na_3VO_4, 10% glycerol, containing freshly added protease and phosphatase inhibitors). The volume of lysis buffer was calculated as 40 mg of tumor/mL. The tissue was homogenized by hand homogenizer for 8 seconds, and then transferred to microcentrifuge tubes and centrifuged at 4°C, 14,000 rpm for 10 minutes. Supernatant was collected and diluted to protein concentration of 1.5 µg/µL. The cell lysate was mixed with 4×SDS and were submit for RPPA assay. The RPPA were performed at the Process Core Facility at MD Anderson (http://bioinformatics.mdanderson.org/OOMPA). In brief, we arrayed these lysates on nitrocellulose-coated FAST slides (Whatman, Inc., Sanford, ME). Slides then were scanned and analyzed to quantitatively measure spot density to generate a fitted curve for each condition. The

fitted curve was plotted with the log_2-concentration of proteins versus spot density. Data presented in the treatment groups reflect fold-change compared to the baseline (i.e., to untreated control group), and analyzed by Ingenuity Pathway Analysis (IPA, Qiagen). Positive fold-change was calculated by dividing each linear value >1.0 by the average control linear value for each antibody tested, while negative fold-change (for linear values <1.0) was also calculated (by using the following formula: [-1/linear fold-change]). The median of the expression fold-change from three individual replica in each condition was plotted in the heatmap. The p values associated with pathways in IPA analysis were calculated using the right-tailed Fisher Exact Test.

Table S1. Peptides used in EphB4 phosphorylation screening

Sequence	Samples	MW (Da)	K_D (nM)
Cyclo(KTNYLFSPNGPIARAWD)	B4-001	1932	4.4
TNYLFSPNGPIA(dR)AW	B4-002	1706	39
*YSA-TNYL-RAW-dimer	B4-003	3950	21
TNY(dL)FSPNGPIARAW	B4-004	1706	65
TNYL(dF)SPNGPIARAW	B4-005	1706	75
TNYLF(dS)PNGPIARAW	B4-006	1706	>1000
TN(dY)LFSPNGPIARAW	BIDEN-AP	1706	7.0
TNYLFSPNGPIARA	B4-008	1518	13
TNYL-RAW		1706	3.1

*YSA = YSAYPDSVPMMS

Table S2. Major pathways and factors regulated by BIDEN-AP

**Top Canonical Pathways (Downregulated)**	**p-value**
Regulation of the EMT pathway	$4.86x10^{-9}$
VEGFR-regulated endothelial cell signaling	$3.16x10^{-8}$
Ovarian cancer signaling	$1.52x10^{-7}$
**Top Regulators (downregulated)**	
EGFR	$3.18x10^{-17}$
VEGFR2	$2.78x10^{-17}$
GAB2	$1.40x10^{-16}$
JAK2	$6.00x10^{-17}$
**Top Regulators (upregulated)**	
TP53	$9.06x10^{-15}$
BAX	$5.42x10^{-7}$
BID	$7.86x10^{-8}$
ATG7	$5.92x10^{-8}$
RICTOR	$5.47x10^{-3}$

These results were obtained from integrated pathway analysis by comparing reverse-phase protein array data from A2780cp20-Luc tumors treated with BIDEN-AP with untreated groups (Control). The p values associated with each pathways in Ingenuity Pathway Analysis were calculated using the right-tailed Fisher Exact Test, and the most significant ones were displayed in this table.

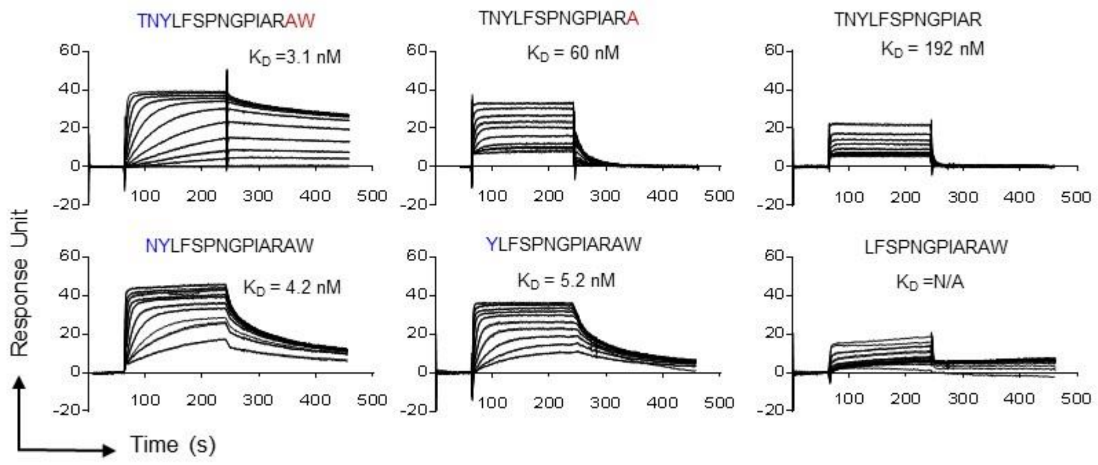

Fig. S1. BIACore sensorgrams of binding of truncated peptides to immobilized rhEphB4. Retention of a peptide on a sensor chip was indicated by a change in response units (RU) over the course of the 180-s injection interval followed by a 210-s washing interval. Peptides in concentrations of 1.6 nM to 800 nM were injected over the rhEphB4 sensor chip, and the magnitude of peptide binding to immobilized EphB4 was recorded.

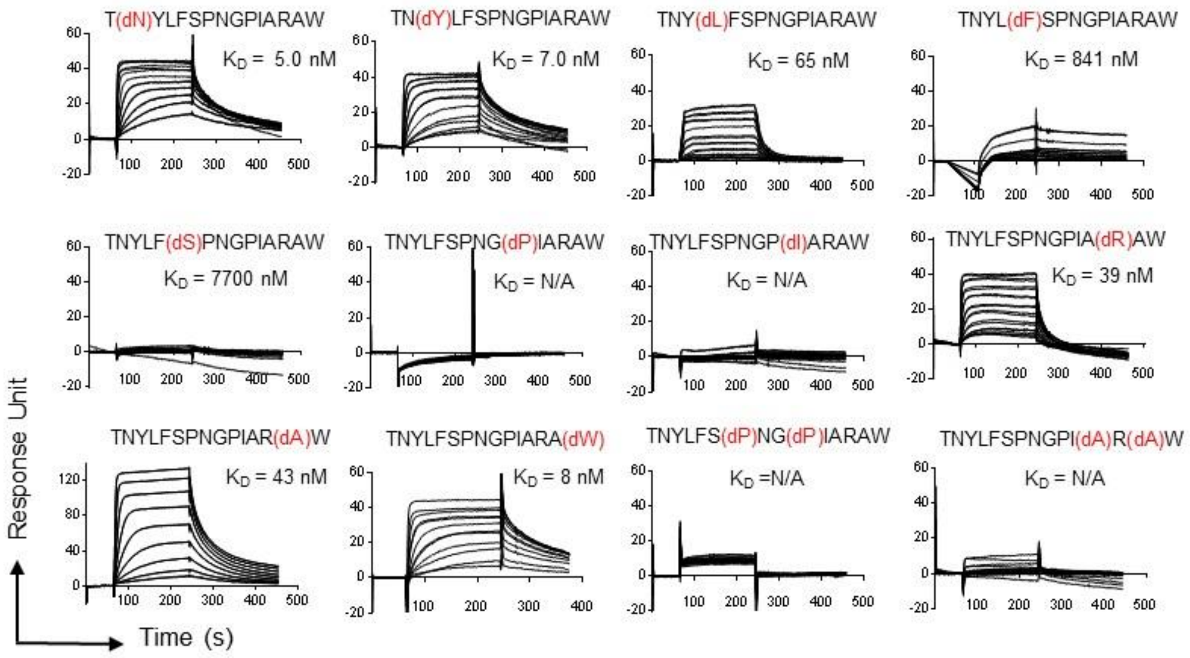

Fig. S2. **BIACore sensorgrams of binding of selected D-substituted TNYL-RAW peptides to immobilized rhEphB4.** Peptides in concentrations of 1.6 nM to 800 nM were injected over the rhEphB4 sensor chips, and the magnitude of peptide binding to immobilized EphB4 in response units (RU) was recorded.

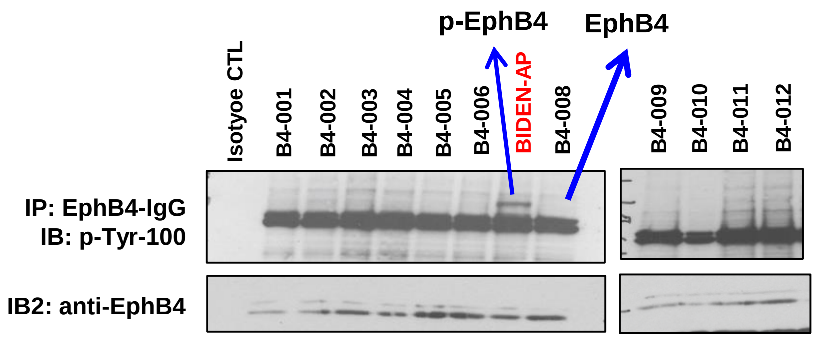

Fig. S3. BIDEN-AP induces EphB4 phosphorylation. HeyA8 ovarian cancer cells were grown to 75% confluence in 6-well plates. Cells were washed and incubated with serum-deprived medium for 12 h. Cells were then treated for 12 h with each test compound at a concentration of 50 nM in complete medium. Cell lysates were subjected to immunoprecipitation (IP) using 10 µL (5 µg) mouse anti-EphB4 receptor antibody and 18 µL protein G beads. After resolving with 4-20% Nu-PAGE gel, the immunoblotting were performed with anti-phosphorylated Tyrosine and anti-EphB4 antibodies . Mouse IgG was included as an isotype control for immunoprecipitation. The uncropped blots were available at Supplemental Figure 11.

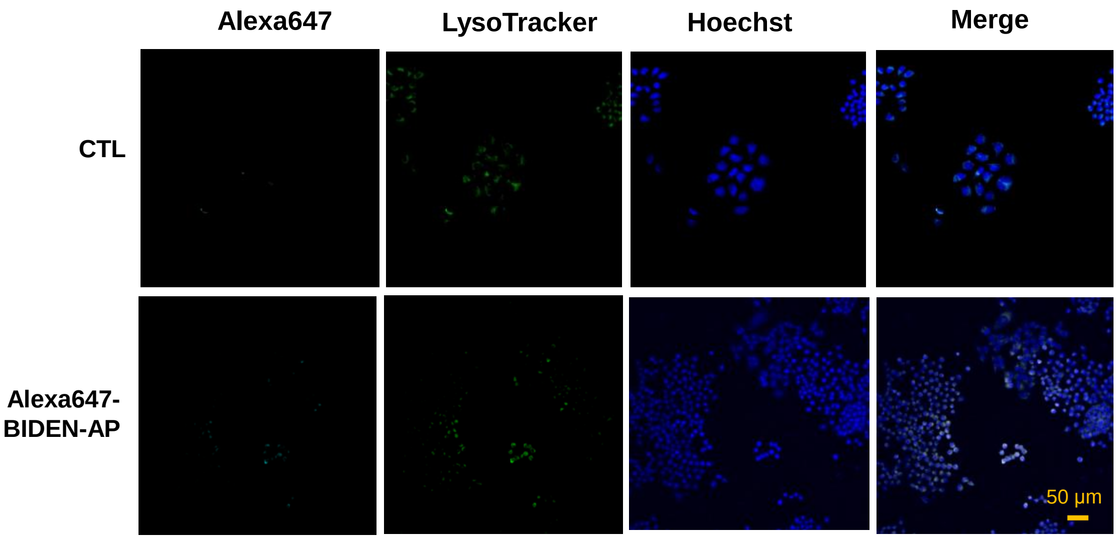

Fig. S4. Alexa647-BIDEN-AP was not internalized in ovarian cancer cells at 4°C. A2780cp20 cells were incubated at 4°C with Alexa647-BIDEN-AP (0.05 μM). The compound was not taken up by the tumor cells at low temperature, suggesting an energy-dependent receptor-mediated endocytic process for Alexa647-BIDEN-AP.

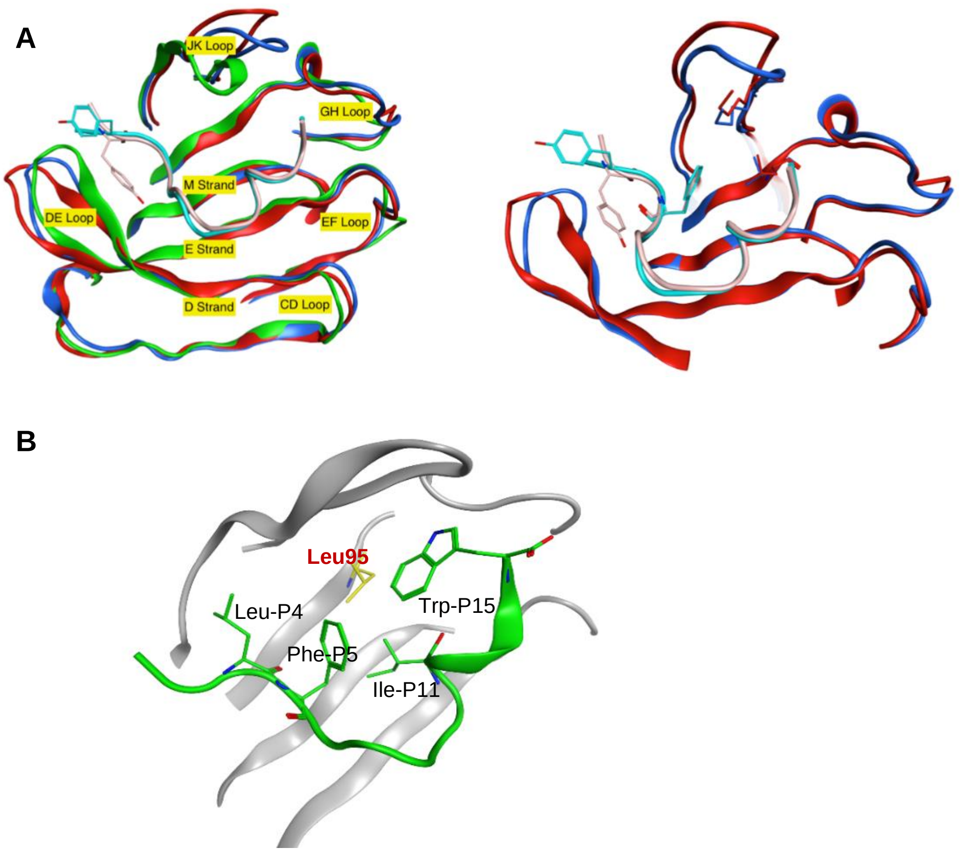

Fig. S5. Superimposition of EphB4 structures. **(A)** The green ribbon shows EphB4 conformation in the presence of the natural ligand EFNB2 (PDB:2HLE), the red ribbon shows conformation with antagonist (PDB:2BBA), and the blue ribbon shows modeled EphB4 conformation bound to BIDEN-AP. Antagonist peptide TNYLA-RAW is shown in light pink and BIDEN-AP in the teal color. **(B)** TNYL-RAW and BIDEN-AP interactions with EphB4. Leu95 of EphB4 could accommodate both peptides through hydrophobic interactions with Leu-P4, Phe-P5, Ile-P11, and Trp-P15. Other EphB receptors would generate steric clash with both peptides and thus would not bind these peptides.

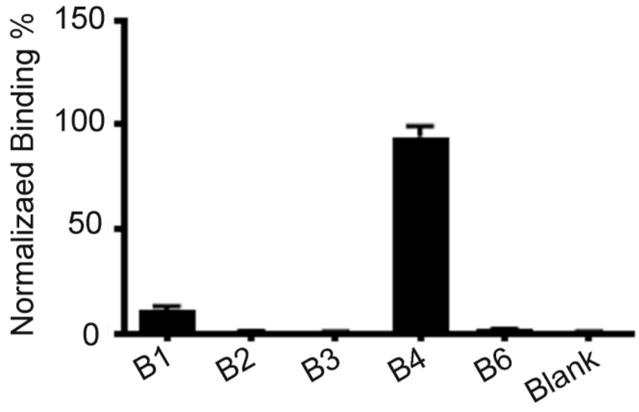

Fig. S6. ELISA binding assay to EphB class receptors. BIDEN-AP selectively bound to EphB4 but not other members in the EphB receptor family. Data are expressed as mean ± SD (n = 3).

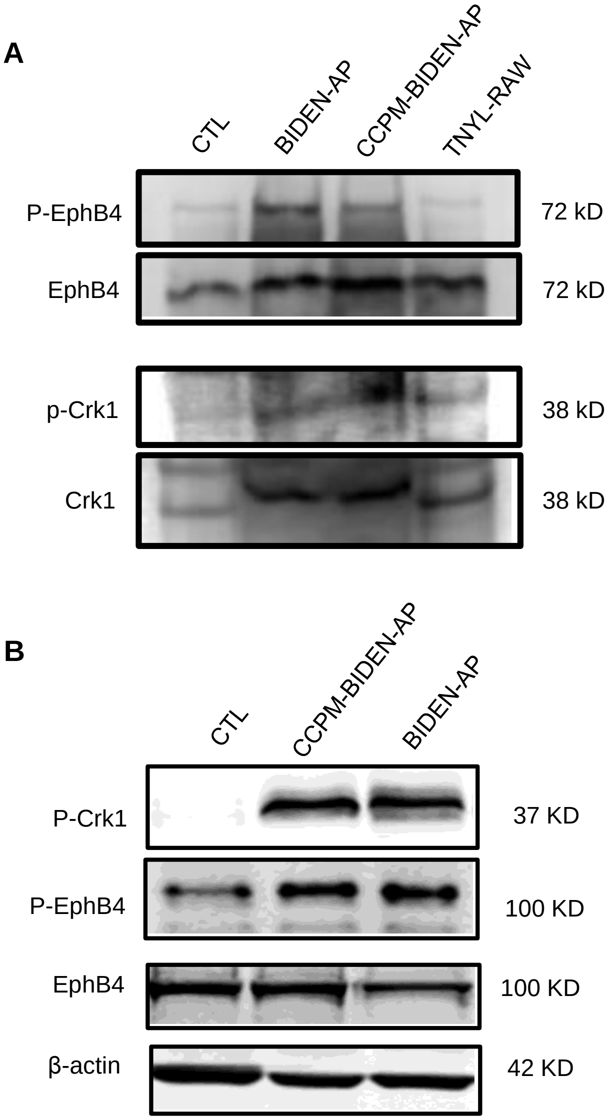

Fig. S7. Both BIDEN-AP and CCPM-BIDEN-AP activate EphB4 and the associated Crk1 in human ovarian cancer cells or lysates from orthotopic ovarian tumors (**A**) A2780cp20 cells were incubated with BIDEN-AP (50 nM), CCPM-BIDEN-AP (50 nM equivalent concentration to BIDEN-AP), or TNYL-RAW (50 nM) for 24 h after the cells had been cultured in serum-free medium for 16 h. Total protein lysates were harvested and subjected to Nupage-SDS separation followed by immunoblotting. (**B**) Immunoprecipitation and immunoblotting for analyzing phosphorylated EphB4 and Crk1 levels in orthotopic A2780cp20 tumors in mice treated with BIDEN-AP or CCPM-BIDEN-AP. CTL, untreated control. The uncropped blots were available at Supplemental Figure 11.

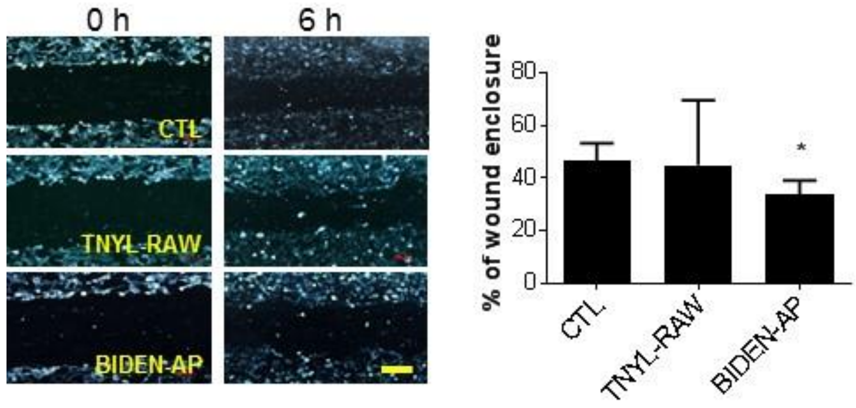

Fig. S8. BIDEN-AP inhibits migration ability of endothelial cells. RF24 cells were treated with BIDEN-AP (50 µM) or TNYL-RAW (50 µM), and subjected to a scratch assay to access cell migration. Scale bar, 200 µm. Mean wound closure was determined for each group. CTL, untreated control. *p<0.05 (compared to CTL; n = 7).

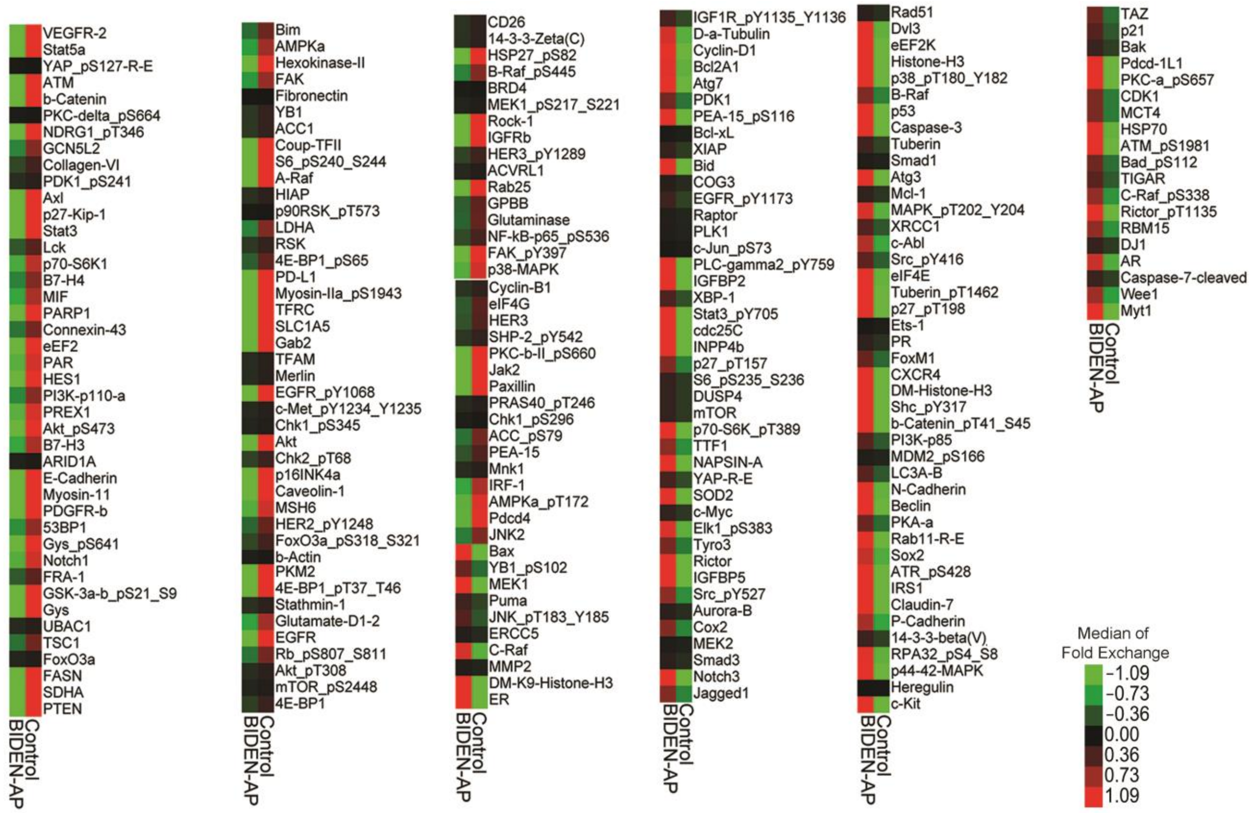

Fig. S9. Heatmap representing 263 array spots obtained from reverse-phase protein array. Three individual A2780cp20-Luc tumors from untreated mice (control) and BIDEN-AP-treated mice (BIDEN-AP) were used in the analysis. The median of the expression fold-change from three individual replica in each condition was plotted in this heatmap.

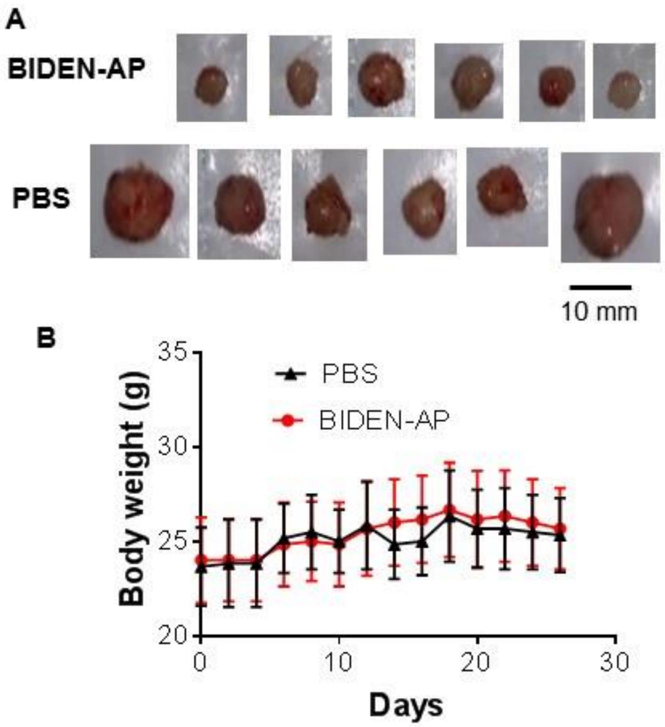

Fig. S10. Antitumor activity of BIDEN-AP against a subcutaneously implanted ovarian cancer PDX model. (**A**) Photographs of excised tumors at the end of a 28-day study period. (B) Body weight change over the course of the study. Data are expressed as mean ± standard deviation.

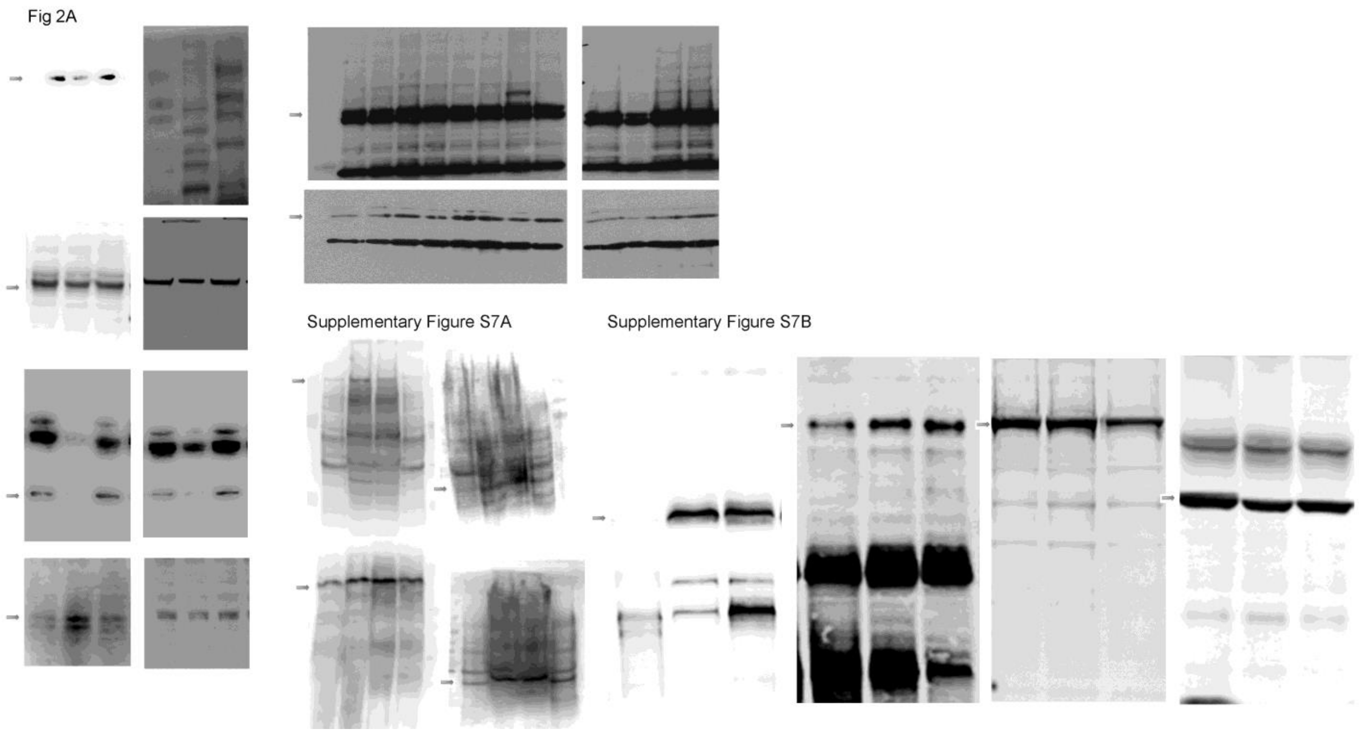

Fig. S11. Original western blot data.
